# Supplementary material for: Spliceosome factors target timeless (tim) mRNA to control clock protein accumulation and circadian behavior in Drosophila
Source: eLife. 2018 Dec 5;7:e39821. doi: 10.7554/eLife.39821 (PMC6281371; doi:10.7554/eLife.39821)
Supplement: Supplementary file 1. [file elife-39821-supp1.docx]

Supplemental Table 1. DAVID pathway analysis of changes in gene expression upon *prp4* downregulation

| **Category** | **Term** | **Gene Count** | **% of genes in**  **the category** | **P-value** | **Q-value**  **(Benjamini)** |
| --- | --- | --- | --- | --- | --- |
| KEGG_PATHWAY | Protein export | 6 | 0.8 | 1.2E-2 | 7.0E-1 |
| KEGG_PATHWAY | Protein processing  in the endoplasmic reticulum | 16 | 2.2 | 1.6E-2 | 5.6E-1 |
| KEGG_PATHWAY | Drug metabolism –  other enzymes | 8 | 1.1 | 3.2E-2 | 6.5E-1 |
| KEGG_PATHWAY | Folate biosynthesis | 5 | 0.7 | 7.7E-2 | 8.6E-1 |
| KEGG_PATHWAY | Metabolic pathways | 69 | 9.3 | 9.7E-2 | 8.6E-1 |
